# H19 lncRNA identified as a master regulator of genes that drive uterine leiomyomas

**DOI:** 10.1038/s41388-019-0808-4

**Published:** 2019-05-15

**Authors:** Tiefeng Cao, Ying Jiang, Zhangsheng Wang, Na Zhang, Ayman Al-Hendy, Ramanaiah Mamillapalli, Amanda N. Kallen, Pinar Kodaman, Hugh S. Taylor, Da Li, Yingqun Huang

**Affiliations:** 1 Department of Obstetrics, Gynecology, and Reproductive Sciences, Yale University School of Medicine, New Haven, CT 06510 USA; 2 Department of Gynecology and Obstetrics, First Affiliated Hospital of Sun Yat-Sen University, Guangzhou, Guangdong 510070 China; 3 Department of Obstetrics, Women’s Hospital, Zhejiang University School of Medicine, Hangzhou, Zhejiang 310006 China; 4 Department of Cardiology, Fifth People’s Hospital of Shanghai, Fudan University, Shanghai, 200240 China; 5 Department of Genetics and Genome Sciences, University of Connecticut Health Center, Farmington, CT 06030 USA; 6 Department of Obstetrics and Gynecology, University of Illinois College of Medicine, Chicago, IL 60612 USA; 7 Center of Reproductive Medicine, Department of Obstetrics and Gynecology, Shengjing Hospital, China Medical University, Shenyang, 110004 China

**Keywords:** Gynaecological cancer, Epigenetics

## Abstract

Uterine leiomyomas or fibroids (UFs) are benign tumors characterized by hyperplastic smooth muscle cells and excessive deposition of extracellular matrix (ECM). Afflicting ~80% of women, and symptomatic in 25%, UFs bring tremendous suffering and are an economic burden worldwide; they cause severe pain and bleeding, and are the leading cause of hysterectomy. Yet, UFs are severely understudied with few effective treatment options available; those that are available frequently have significant side effects such as menopausal symptoms. Recently, integrated genome-scale studies have revealed mutations and fibroid subtype-specific expression changes in key driver genes, with *MED12* and *HMGA2* together contributing to nearly 90% of all UFs, but their regulation of expression is poorly characterized. Here we report that the expression of H19 long noncoding RNA (lncRNA) is aberrantly increased in UFs. Using cell culture and genome-wide transcriptome and methylation profiling analyses, we demonstrate that H19 promotes expression of *MED12*, *HMGA2*, and key ECM-remodeling genes via multiple mechanisms including a new class of epigenetic modification by TET3. Our results mark the first example of an evolutionarily conserved lncRNA in pathogenesis of UFs and regulation of TET expression. Given the link between a H19 single-nucleotide polymorphism (SNP) and increased risk and tumor size of UFs, and the existence of multiple fibroid subtypes driven by key pathway genes regulated by H19, we propose a unifying mechanism for pathogenesis of uterine fibroids mediated by H19 and identify a pathway for future exploration of novel target therapies for uterine leiomyomas.

## Introduction

Uterine fibroids (UFs) or leiomyomas are benign tumors arising from the smooth muscle compartment of the uterus. Up to 60% of reproductive-aged women and 80% of all women suffer from fibroids during their lifetime [1]. Fibroids are associated with pelvic pain, excessive bleeding, infertility, and pregnancy complications, and they are the leading indication for hysterectomy. Despite the high prevalence and being huge heath and economic burdens, few effective treatment options exist for UFs. This is largely due to the limited understanding of the molecular basis underlying the etiology and pathogenesis of the disease.

UFs are characterized by hyperplastic smooth muscle cells and excessive deposition of extracellular matrix (ECM) [2, 3]. Many factors contribute to the development of fibroids, including steroid hormones, growth factors, and genetic traits. Mutations in mediator complex subunit 12 (*MED12*) have been frequently found in fibroids [4]. A gain-of-function mutation of a common *MED12* variant was able to drive fibroid formation and cause genomic instability in mice, suggesting a causative role of *MED12* mutations in fibroids [5]. Further, overexpression of wild-type *MED12* promotes proliferation of leiomyoma cells [6]. Other factors that have been implicated in fibroids include high-mobility group AT-hook 2 (HMGA2), transforming growth factor (TGF)-β receptor 2 (TGFBR2), thrombospondin 1 (TSP1), Rho GTPase-activating protein 26 (ARHGAP26, also called GRAF1), secreted protein acidic and rich in cysteine (SPARC), and Ten eleven translocation (TET) family proteins. Although MED12 and HMGA2 have been implicated in smooth muscle hyperplasia, TGFBR2, TSP1, GRAF1, and SPARC are associated with abnormal ECM remodeling [3, 7–10].

Canonical TGF-β signaling requires TGF-β, TGFBR2, and TGFBR1, and Smad proteins (Smad2, Smad3, and Smad4). TGF-β is secreted as a latent precursor that must be converted into a biologically active form by a variety of mechanisms including proteolytic cleavage by TSP1. Activated TGF-β binds to TGFBR2, which recruits and activates TGFBR1. TGFBR1 then phosphorylates Smad2 and Smad3, which complex with Smad4 and translocate into the nucleus to drive transcription of profibrotic molecules leading to excessive ECM production. Thus, pathological activation of TGF-β signaling plays a critical role in the development and progression of fibrosis (reviewed in refs. [8, 11, 12]).

The TET proteins (TET1, TET2, and TET3) are a newly discovered family of DNA demethylases that act to oxidize 5-methylcytosine to generate 5-hydroxymethylcytosine (5hmC), which is subsequently converted to unmethylated cytosine via the base excision repair pathway, leading to DNA demethylation and gene activation [13–15]. Importantly, elevated expressions of TET1 and TET3 have been detected in fibroids as compared with matched myometrium. Small interfering RNA (siRNA) knockdown of either TET1 or TET3 leads to decreased proliferation of primary leiomyoma cells, suggesting a potentially important role of TETs in the pathogenesis of fibroids [16]. Recent integrative genome-scale studies of fibroids harboring different genetic alterations, including *MED12* mutations and *HMGA2* rearrangements, have uncovered fibroid subtype-specific gene expression signatures, with *MED12* and *HMGA2* being the most common driver genes that together contribute to 80–90% of all fibroids [17].

The evolutionarily conserved H19 long noncoding RNA (lncRNA) is highly expressed in placentas and fetal tissues, and is strongly downregulated in most adult tissues [18]. However, H19 expression is aberrantly elevated in fibrotic conditions in multiple organs including the liver, lung, and kidney [19–21]. As a multi-functional lncRNA, H19 is polyadenylated and localizes predominantly in the cytoplasm. We have previously reported the H19/let-7 axis where H19 acts as a molecular sponge for microRNA let-7, thereby reducing its bioavailability and preventing it from inhibiting target gene expression at the posttranscriptional level [22]. In this report we show that H19 expression is significantly increased in fibroids as compared with normal myometrium, and that H19 functions to promote leiomyoma cell proliferation and expression of *MED12*, *HMGA2*, *TET3*, and ECM-remodeling genes. Mechanistically, we demonstrate that H19 regulates gene expression via both posttranscriptional and TET3-dependent epigenetic mechanisms. Taken together with the notion that a single-nucleotide polymorphism (SNP) of H19 is linked to increased risk and tumor size of fibroids [9, 23], our results suggest a critical role of H19 in the pathogenesis of UFs.

## Results

### H19 expression is aberrantly elevated in fibroids

**Fig. 1:**
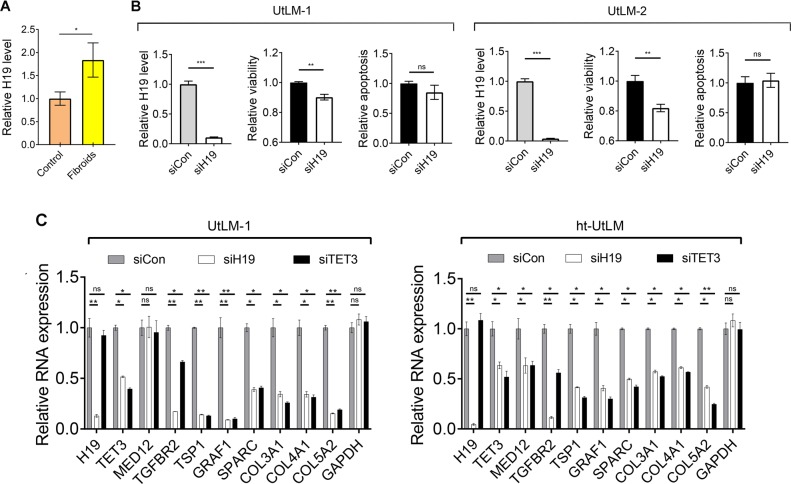
H19 promotes cell proliferation and expression of fibroid-promoting genes. **a** RT-qPCR results of H19 expression in fibroids and matched myometrium (control). *n* = 30. **b** Primary leiomyoma cells derived from fibroid tumors of two patients were transfected with control siRNA (siCon) or H19-specific siRNA (siH19), followed by RT-qPCR (left panels), cell viability (middle panels), and caspase-3/7 activity (right panels) analyses at 48 h post transfection. *n* = 3–5. **c** UtLM-1 or ht-UtLM cells were transfected with siCon, siH19, or a TET3-specific siRNA (siTET3). RNA levels were measured by RT-qPCR 48 h later. *n* = 3, one-way ANOVA with Dunnett’s post test. All data are representative of at least two independent experiments and are presented as mean ± SEM. *p < 0.05, **p < 0.01. ns, not statistically significant

UFs are a fibrotic disorder characterized by excessive ECM deposition [2, 24]. As H19 has been implicated in the fibrosis of the liver, lung, and kidney [19–21], we tested whether H19 expression is altered in UFs. Thus, paired fibroids (Fibroids) and myometrial tissues (Control) were collected from women at the proliferative stage of the menstrual cycle, who underwent a hysterectomy or myomectomy for fibroids. RNAs were extracted from the tissue samples and H19 expression levels were determined by reverse transcription and quantitative real-time PCR (RT-qPCR) analysis. A statistically significant increase in H19 expression in fibroids vs. matched myometrium was observed (Fig. 1a), suggesting a role of H19 in UFs.

### H19 promotes proliferation of leiomyoma cells

To assess the role of H19 in cell growth, H19 knockdown experiments were performed on primary human leiomyoma cells (UtLM-1 and UtLM-2) derived from the fibroid tumors of two patients. Thus, H19-specific siRNA (siH19 [25],) or control siRNA (siCon) were transfected into cells, followed by analyses of cell viability (as a readout for cell proliferation) and caspase-3/7 activity (as a readout for cell apoptosis) at 48 h post transfection. When H19 was downregulated (Fig. 1b, left panels), cell viability decreased (middle panels) without affecting apoptosis (right panels), suggesting that H19 positively affects leiomyoma cell proliferation.

### H19 regulates expression of fibroid-promoting genes

To begin to elucidate the mechanism of H19-mediated regulation, H19 knockdown studies were carried out in normal human primary uterine smooth muscle cells. RNAs were extracted at 48 h post transfection and subjected to high-throughput deep sequencing (RNA-sequencing). Results showed that among the numerous genes whose expressions were downregulated in siH19-transfected cells were those involved in 5hmC epigenetic regulation, TGF-β signaling, ECM remodeling, and cell growth (Supplementary Table 1 and GEO accession number GSE110557). Decreased expression of genes (*TET3*, *MED12*, *TGFBR2*, *TSP1*, *GRAF1*, *SPARC*, *COL3A1*, *COL4A1*, and *COL5A2*) previously implicated in fibroids was also observed following H19 knockdown in primary human leiomyoma cells (Fig. 1c, left panel, compare white bars with gray bars across all columns), as well as in ht-UtLM, an immortalized human leiomyoma cell line [26] (Fig. 1c, right panel). Remarkably, when TET3 was downregulated using siRNA (siTET3) (Fig. 1c, second columns from left, compare black bars with gray bars), results similar to those of H19 knockdown were obtained (Fig. 1c, compare black bars with gray bars across all columns). The lack of effects of H19 or TET3 knockdown on *MED12* expression in ht-UtLM cells was likely due to a much lower endogenous level of H19 as compared with UtLM cells (Supplementary Fig. 1). To determine whether decreased mRNA expression leads to decreased protein levels, western blotting analysis was conducted using UtLM cells. Results showed that when H19 (Fig. 1c, first column from left, compare white bar with gray bar) or TET3 (Fig. 1c, second column from left, compare black bar with gray bar; Supplementary Fig. 2A) was downregulated, the protein levels of MED12 (Supplementary Fig. 2B), TGFBR2 (Supplementary Fig. 2C), TSP1 (Supplementary Fig. 2D), GRAF1 and SPARC (Supplementary Fig. 2E), COL5A2, COL4A1, and COL3A1 (Supplementary Fig. 2F), were all decreased, consistent with mRNA results (Fig. 1c). Collectively, these results suggested that H19 positively regulates expression of a subset of fibroid-promoting genes including *TET3*, which appears to be also an important downstream mediator of H19.

### H19 regulates HMGA2 and TET3 expression via the H19/let-7 axis

**Fig. 2:**
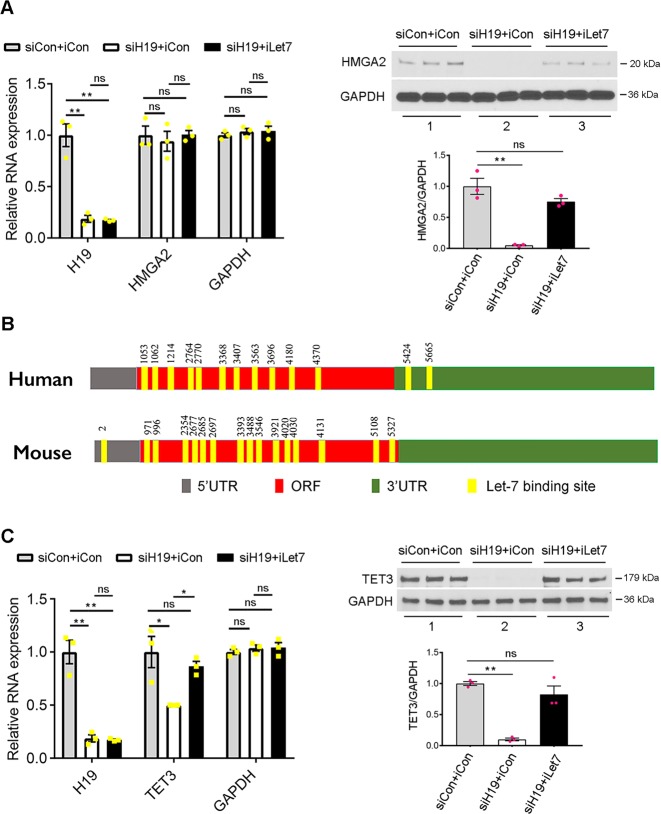
H19 regulates expression of HMGA2 and TET3 posttranscriptionally via let-7. **a**, **c** UtLM cells were transfected with siCon + iCon (miRNA inhibitor control), siH19 + iCon, or siH19 + iLet7; RNA and proteins were isolated 48 h later and analyzed by RT-qPCR (left panels) or western blotting (right panels). *n* = 3, one-way ANOVA with Tukey’s post- test. Data are representative of two independent experiments and are presented as mean ± SEM. *p < 0.05, **p < 0.01. ns, not statistically significant. **b** Schematics of human and mouse TET3 mRNAs, with numbers on top depicting positions of let-7-binding sites relative to the transcriptional start sites. Figures are not drawn to scale

Our previous work documented the H19/let-7 axis where H19 acts as a “molecular sponge” to reduce the bioavailability of let-7: H19 contains multiple let-7-binding sites that sequester let-7 and prevent it from binding to target mRNAs [22]. Binding of let-7 to complementary sequences in target mRNAs results in translational repression and/or mRNA degradation. Therefore, let-7 action can lead to decreased protein levels with or without altering mRNA levels [27]. HMGA2 contains let-7-binding sites in its 3′-untranslated region and is a validated target of let-7 [28, 29]. In our previous studies of human endometrial cancer and ovarian cancer cells, we showed that H19 positively regulates *HMGA2* expression via the H19/let-7 axis [30]. As *HMGA2* is among the key driver genes in leiomyomas [17], we tested whether H19 regulates its expression in leiomyoma cells. Thus, H19 knockdown experiments in combination with a let-7-specific inhibitor (iLet7) [25, 31] were carried out in UtLM cells, followed by analysis of *HMGA2* expression. iLet7 are chemically modified, single-stranded nucleic acids that bind to let-7 specifically and block its activity. The effect of H19 knockdown (i.e., downregulation of *HMGA2*) would be abolished in the presence of iLet7, which acts to neutralize let-7 released from H19 sequestration. Indeed, when H19 was knocked down in the absence (Fig. 2a, left panel, left column, compare white bar with gray bar) or presence (compare black bar with gray bar) of iLet7, there was no change in HMGA2 mRNA levels (middle column). However, when H19 was knocked down in the absence of iLet7, HMGA2 protein level was significantly decreased (Fig. 2a, right panel, top blots, compare lane 2 with lane 1; bottom graph, compare middle bar with left bar), which was restored to the control level when iLet7 was present (top blots, compare lane 3 with lane 1; bottom graph, compare right bar with left bar). This suggested that H19 deficiency led to enhanced let-7 action, enabling it to repress expression of *HMGA2* at the translational level without altering HMGA2 mRNA levels. Thus, we concluded that in primary leiomyoma cells H19 promotes *HMGA2* expression at the translational level by reducing the bioavailability of let-7.

To determine how H19 regulates *TET3* expression, we performed bioinformatics analysis and predicted multiple let-7-binding sites in the coding regions of both human and mouse TET3 mRNAs (Fig. 2b). This suggested that H19 may promote *TET3* expression by sequestering let-7. Thus, H19 knockdown experiments in the absence and presence of iLet7 were performed in UtLM cells, followed by analysis of *TET3* expression. H19 knockdown led to decreased *TET3* expression at both mRNA (Fig. 2c, left panel) and protein (right panel) levels, but the expression was restored to control levels in the presence of iLet7. These results suggested that TET3 is a novel target of let-7, and that H19 regulates *TET3* expression via the H19/let-7 axis.

### TET3 binds to target gene promoters and regulates DNA methylation and histone modifications

**Fig. 3:**
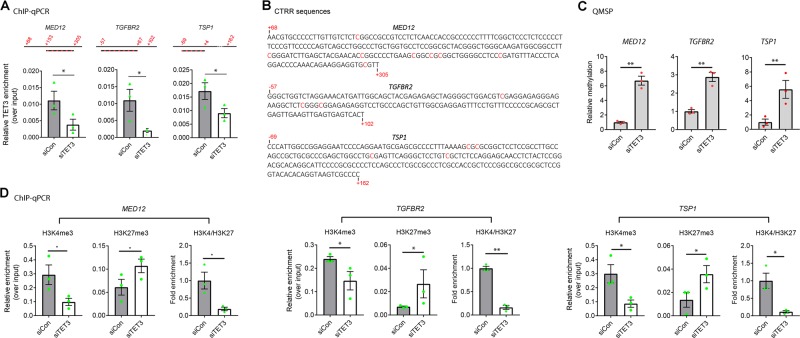
TET3 affects DNA methylation and histone modifications of the MED12, TGFBR2, and TSP1 promoters. **a** UtLM cells were transfected with siCon or siTET3 for 48 h, followed by ChIP-qPCR analysis. Data are presented as mean relative TET3 enrichment over input. *n* = 3. Red numbers indicate nucleotide positions relative to the transcriptional start sites, with PCR products depicted as red-stripped bars. **b** Sequences of critical transcription regulatory regions (CTRR) of *MED12*, *TGFBR2*, and *TSP1*. The differentially methylated cytosine residues are marked in red. The red numbers mark the positions of the indicated nucleotides relative to the transcriptional start sites. **c** UtLM cells were transfected with siCon or siTET3 for 48 h, followed by QMSP analysis. *n* = 3. **d** UtLM cells were transfected with siCon or siTET3 for 48 h, followed by ChIP-qPCR analysis. Data are presented as mean relative enrichment over input. *n* = 3. All data are representative of at least two independent experiments and are presented as mean ± SEM. *p < 0.05, **p < 0.01

As TET3 positively regulates expression of fibroid-promoting genes such as *MED12*, *TGFBR2*, and *TSP1* among others (Fig. 1c and Supplementary Fig. 2), we sought to test the possibility of a direct interaction between TET3 and target genes. Thus, we performed chromatin immunoprecipitation coupled with qPCR (ChIP-qPCR) experiments using a TET3-specific antibody to immunoprecipitate protein–DNA complexes from UtLM cells transfected with siCon or siTET3 for 48 h and qPCR amplified the critical transcriptional regulatory regions (CTRRs) of the *MED12* [32], *TGFBR2* [33], and *TSP1* [34] promoters. In TET3 knockdown cells, binding of TET3 to the respective promoters was significantly reduced as compared with control cells, consistent with physical interactions of TET3 with these promoters (Fig. 3a).

It is well established that TET proteins promote DNA demethylation leading to alteration of chromatin states. First, we assessed TET3-induced DNA methylation changes using ht-UtLM cells. Cells were transfected with siTET3 (or siCon as a control), and genomic DNA was extracted 48 h later and subjected to single-nucleotide resolution genome-wide DNA methylation profiling. As expected, following TET3 knockdown we observed extensive DNA methylation changes relative to siCon-treated cells, with some genes showing increased methylation, others showing decreased methylation, and a third group with no significant change (GEO accession number GSE117190). Remarkably, we observed increased methylation of the CpGs within the CTRRs of the *MED12*, *TGFBR2*, and *TSP1* promoters (Fig. 3b, differentially methylated cytosine residues highlighted in red). This was further confirmed in UtLM cells using quantitative methylation-specific PCR (QMSP) analysis (Fig. 3c), our previously established method [35, 36]. The QMSP primers were designed based on the differentially methylated cytosine residues (Fig. 3b and Supplementary Table 2). Collectively, these results suggested that binding of TET3 to *MED12*, *TGFBR2*, and *TSP1* induces promoter demethylation.

Next, we tested whether TET3 knockdown affects chromatin states. UtLM cells were transfected with siCon or siTET3, followed by ChIP-qPCR, immunoprecipitating with antibodies specific for the H3K4me3 (active) or H3K27me3 (inactive) marks, and amplifying the CTRRs of *MED12*, *TGFBR2*, and *TSP1*. ChIP analysis showed that TET3 knockdown significantly decreased H3K4me3 (Fig. 3d, left columns) and increased H3K27me3 (middle columns) association with all three promoters, such that the ratios of H3K4me3/H3K27me3 decreased by five- to eightfold (right columns). These results suggested that TET3 knockdown promotes a heterochromatin conformation and diminishes chromatin accessibility at the *MED12*, *TGFBR2*, and *TSP* promoter regions.

### H19 and TET3 expression positively correlates with expression of key fibroid-promoting genes in vivo

**Fig. 4:**
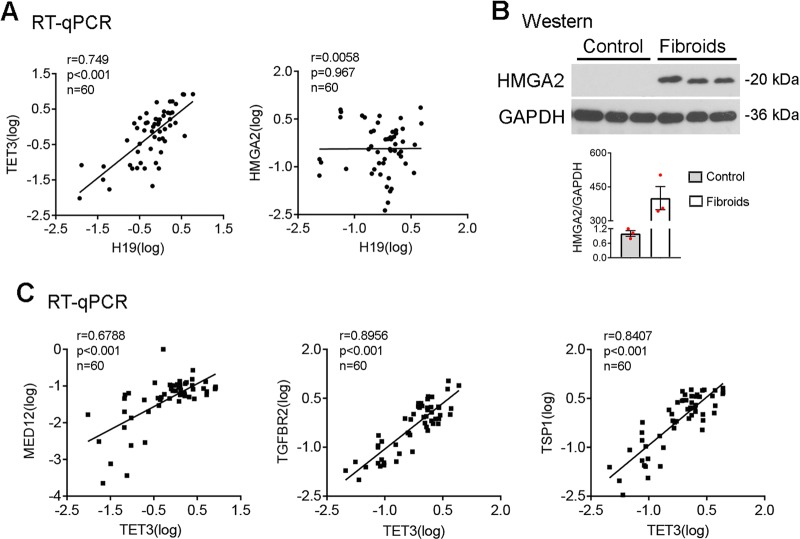
H19 and TET3 co-express with fibroid-promoting genes in vivo. **a**, **c** RT-qPCR analyses were performed on RNAs extracted from human fibroids and matched myometrium tissues. Spearman’s correlation showed positive correlations between expression of *H19* and *TET3* (**a**, left panel), as well as *TET3* and its target genes *MED12*, *TGFBR2*, and *TSP1* (**c**) in a statistically significant manner. No correlation between expression of H19 and HMGA2 at the RNA level was detected (**a**, right panel). Spearman’s correlation coefficient, *p*-values, and sample numbers are presented. **b** Results of western blotting analysis of HMGA2 in human fibroids and matched myometrium. *n* = 3. Data are representative of two independent experiments and are presented as mean ± SEM

To provide evidence supporting the in vivo roles of H19 and TET3 in fibroids, we performed RT-qPCR analysis on RNA samples derived from fibroids and matched myometrium. There was a positive correlation in expression between H19 and TET3, and the trend was statistically significant (Fig. 4a, left panel), consistent with our in vitro data showing that H19 positively regulates TET3 expression (Fig. 2c). No correlation between H19 and HMGA2 at the RNA level was detected (Fig. 4a, right panel). However, western blotting analysis showed a clear increase at the HMGA2 protein level in fibroids vs. normal myometrium (Fig. 4b), consistent with our in vitro observation that H19 promotes HMGA2 expression at the translational level (Fig. 2a). Next, we analyzed expression of *TET3* and its target genes *MED12*, *TGFBR2*, and *TSP1* in fibroids and matched myometrium. As seen in Fig. 4c, *TET3* expression was positively correlated with expression of all three target genes. These results corroborated our in vitro findings that TET3 knockdown in primary leiomyoma cells decreased expression of *MED12*, *TGFBR2*, and *TSP1* (Fig. 1c, left panel), and that TET3 regulates their expression at the epigenetic level (Fig. 3). Taken together, our results strongly support the in vivo roles of H19 and TET3 in regulation of expression of key fibroid-promoting genes.

### Steroid hormones upregulate fibroid-promoting genes in a H19-dependent manner

**Fig. 5:**
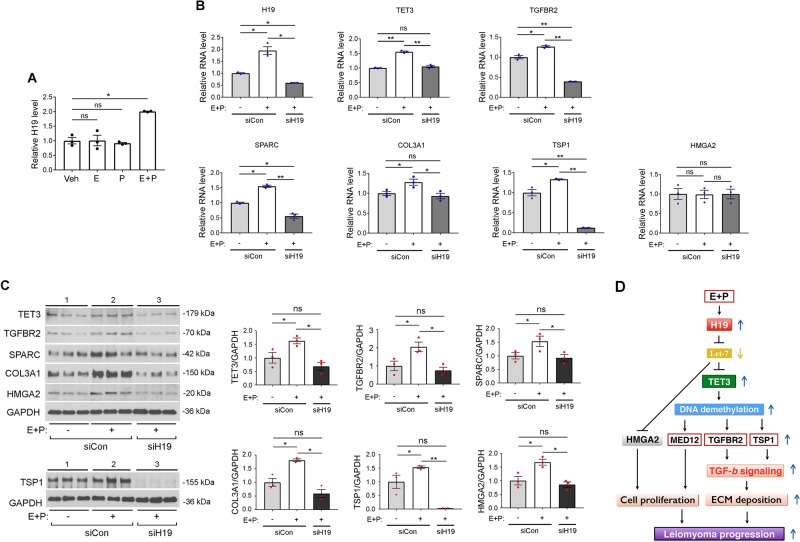
Progesterone and estradiol upregulate fibroid-promoting genes in a H19-dependent manner. **a** ht-UtLM cells were treated with Vehicle (Veh, as a negative control), estradiol (E), progesterone (P), or E and P together for 24 h. RNAs were extracted and H19 levels were determined by RT-qPCR analysis. *n* = 3, one-way ANOVA with Dunnett’s post test. **b** ht-UtLM cells were transfected with siCon or siH19 for 24 h, followed by addition of Veh (−) or E + P (+) for an additional 24 h. RNA levels of the indicated genes were determined by RT-qPCR analysis. *n* = 3, one-way ANOVA with Tukey’s post test. **c** Protein levels were determined by western blot analysis, with quantifications presented on the right. Each group was loaded in triplicate. *n* = 3. In **c**, TSP1 protein was isolated at 72 h post transfection. *n* = 3, one-way ANOVA with Tukey’s post test. **d** A proposed model. All data are representative of at least two independent experiments and are presented as mean ± SEM. *p < 0.05, **p < 0.01. ns, not statistically significant

We treated ht-UtLM cells that express a low level of endogenous H19 (Supplementary Fig. 1) with estradiol (E), progesterone (P), or E + P, and assessed H19 expression 24 h later. Although exposing the cells to E or P alone did not affect H19 expression, a combined treatment with both E and P significantly upregulated H19 (Fig. 5a). To determine whether E + P regulate fibroid-promoting genes and whether the effects are influenced by H19, ht-UtLM cells were treated with E + P in combination with H19 knockdown and a subset of H19-regulated genes were tested. E + P increased the expression of *H19*, *TET3*, *TGFBR2*, *SPARC*, *COL3A1*, and *TSP1*, both at the levels of mRNA (Fig. 5b, compare middle bars with left bars) and protein (Fig. 5c, left panels, compare lanes 2 with lanes 1; right panels, compare middle bars with left bars), and this effect was abolished when H19 was downregulated (Fig. 5b, compare right bars with middle and left bars; c, left panels, compare lanes 3 with lanes 2 and 1; right panels, compare right bars with middle and left bars). Although E + P and H19 knockdown did not affect HMGA2 at the mRNA level (Fig. 5b), they did at the protein level (Fig. 5c), further supporting the notion that H19 regulates *HMGA2* expression at the translational level (Fig. 2a). The fact that H19 knockdown to below basal level (Fig. 5b, compare right bar with left bar) abrogated both E + P- and basal H19-induced effects (compare right bars with left bars; c, left panels, compare lane 3 with lane 1; right panels, compare right bars with left bars) strongly suggests that the E + P-induced expression of fibroid-promoting genes is H19-dependent.

## Discussion

UFs are highly heterogeneous in terms of symptoms, histopathology, treatment requirements, and clinical outcomes. Emerging evidence suggests that genetic alterations and subtype-specific gene expression changes underlie the pathogenesis and heterogeneity of UFs. *MED12* is mutated in 70% of fibroids [4] and a gain-of-function mutation of *MED12* induces fibroid formation and genomic instability in mice [5]. Furthermore, overexpression of wild-type *MED12* promotes proliferation of leiomyoma cells [6]. Together, these findings strongly point to a causal role of *MED12* in fibroids. Another gene that has garnered much attention is *HMGA2*, a well-studied oncogene [28], due to its frequent rearrangements and overexpression in fibroids [37]. Notably, genome-wide analyses of fibroids harboring different genetic alterations have revealed fibroid subtypes with distinct driver pathway genes, and *MED12* and *HMGA2* have been found to be the most common driver genes that together account for nearly 90% of all fibroids [17, 37]. Remarkably, we find that H19 acts as an upstream regulator of both *MED12* and *HMGA2*. We show that H19 regulates their expression via distinct mechanisms: it promotes *HMGA2* expression posttranscriptionally by reducing the bioavailability of let-7 (Fig. 2), whereas enhancing *MED12* expression epigenetically via the action of the DNA demethylase TET3, which we identify as a novel target of let-7-mediated regulation (Fig. 5d).

The most striking characteristic of UFs is the deposition of excessive amounts of ECM, whose crucial role in the pathogenesis of UFs has increasingly been recognized. Excessive ECM accumulation contributes significantly to the growth and stiffness of fibroid tumors. In addition to providing structure to the fibroid, the ECM actively participates in various biochemical signaling processes responsible for cell adhesion, proliferation, survival, differentiation, and migration. The ECM also serves as a reservoir for growth hormones and soluble profibrotic factors that promote tumor growth. Thus, ECM is a potential therapeutic target for UFs (reviewed in ref. [38]). Many signal transduction pathways affect ECM production and remodeling, with TGF-β being the key driver [11, 12]. We show that H19 promotes TGF-β signaling by upregulating expression of *TGFBR2* and *TSP1* via the TET3-mediated epigenetic mechanism (Fig. 5d). The decreased expression of ECM component genes *GRAF1*, *SPARC*, *COL3A1*, *COL4A1*, and *COL5A2* in addition to TGF-β pathway genes *TGFBR2* and *TSP1* (Fig. 1 and Supplementary Fig. 2) following H19 or TET3 knockdown further supports the notion that H19 acts through TET3 to promote TGF-β signaling and ECM production (Fig. 5d). The critical role of altered H19 expression, TGF-β signaling, and ECM deposition in the pathogenesis of UFs is further underscored by findings from genome-wide association studies revealing SNPs in *H19*, *TGFBR2*, and *GRAF1* that affect both the risk and tumor size of UFs [9, 23].

Despite the prevalence and enormous medical and economic impact of UFs, there are few options for the treatment of UFs and their associated symptoms. Hysterectomy, which prematurely ends a women’s reproductive life, remains a predominant method for treating UFs. The currently available non-surgical interventions include GnRH analogs, which cause prolonged suppression of pituitary gonadotropins but are not Food and Drug Administration-approved for long-term use and cause significant side effects including menopausal symptoms, and progesterone receptor modulators, which temporally relieve symptoms but are not available in the United States and have been associated with liver failure in Europe [2, 3]. The existence of multiple fibroid subtypes driven by distinct pathway genes has further complicated treatment decisions and effectiveness. Our identification of H19 as a master regulator of key driver genes including *MED12*, *HMGA2*, and ECM-remodeling genes (Fig. 5d) suggests a potentially unifying mechanism mediated by H19. Finally, we show that progesterone and estradiol in combination (but not alone) induce fibroid-promoting gene expression in a H19-dependent manner (Fig. 5). This is intriguing, because in a leiomyoma xenograft mouse model, a combined treatment of estradiol and progesterone induced tumor growth but estradiol alone did not [39]. The same study also showed that estradiol induced expression of progesterone receptors in leiomyoma cells [39]. It will be important and fascinating to test in the future whether targeting H19 leads to inhibition of UFs without inducing a menopause-like state.

## Materials and methods

### Materials

Antibodies for TET3 (GeneTex, GTX121453; used at a dilution of 1/500), TGFBR2 (Abcam, ab184948; used at a dilution of 1/1000), TSP1 (Abcam, ab85762; used at a dilution of 1/500), MED12 (Novus Biological, NB100–2357; used at a dilution of 1/500), HMGA2 (Proteintech, 20795–1-AP; used at a dilution of 1/500), GRAF1 (Cell Signaling, 8802; used at a dilution of 1/500), SPARC (Cell Signaling, 8725; used at a dilution of 1/500), COL3A1 (LS-Bio, LS-C159386; used at a dilution of 1/1000), COL4A1 (LS-Bio, LS-C100552; used at a dilution of 1/500), COL5A2 (Origene, TA809611; used at a dilution of 1/500), and GAPDH (Abcam, ab128915; used at a dilution of 1/10000) were purchased. Human H19 siRNA (siH19) and control siRNA (siCon) were previously described [25]. siTET3 (Ambion, 4392420/s47239), estradiol (E8875, Sigma), and progesterone (P0130, Sigma) were purchased. Estradiol and progesterone were dissolved in dimethyl sulfoxide and used at a final concentration of 10^−8^ M.

### Tissue sample collection

Paired fibroid and myometrial tissues were collected from 30 women (age = < 50 years, premenopausal, *n* = 20; age > 50 years, postmenopausal, *n* = 10), who had a hysterectomy or myomectomy for UFs in Shengjing Hospital of China Medical University between December 2017 and January 2018. Ethics approval for this study was obtained from the Institutional Review Board at China Medical University and all patients signed informed consent. No women received hormones within 3 months before surgery. For premenopausal women, fibroid and myometrial tissues were collected at the proliferative stage of the menstrual cycle. Normal myometrium tissues were taken at a distance of 2 cm from adjacent fibroids. When multiple fibroids were present, a sample was taken from the body of the largest fibroid. Fibroids were not selected according to their locations (intramural, subserosal, or submucosal); however, degenerative fibroids were excluded. Tissues were snap frozen in liquid nitrogen and stored at 80 °C until use.

### Tissue real-time PCR array

Frozen tissue samples were homogenized in liquid nitrogen, followed by RNA extraction using TRIzol Reagent (15596026, ThermoFisher Scientific) according to the manufacturer’s protocol. DNA contamination was removed by DNase I digestion (AM2222, ThermoFisher Scientific). Total RNA was reverse-transcribed using the PrimeScript™ RT Reagent Kit with gDNA Eraser (TaKaRa, Dalian, China) and amplified by GoTaq® qPCR Master Mix (Promega, Madison, WI, USA) in the ABI ViiA 7 Real-time PCR system (Applied Biosystems, USA). The threshold cycle (Ct) values of each sample were used in the post-PCR data analysis. Gene expression levels were normalized against GAPDH. Sequences of the real-time PCR primers are listed in Supplementary Table 2.

### Primary cell isolation and culture

Fibroid tissues were minced and digested in Hanks’ balanced salt solution containing 1% penicillin, 1% streptomycin, 1% collagenase (Sigma-Aldrich: COLLD-RO ROCHE 11088866001), and 0.01% deoxyribonuclease I (Sigma-Aldrich 10104159001) at 37 °C for 40 min, with gentle vortexing every 10 min. Dispersed cells were filtered through Fisherbrand Sterile 70 μm Nylon Mesh Cell Strainers (Fisher Scientific, 22–363–548). The resulting cells in single cell suspension were seeded onto 10 cm tissue culture dishes in Dulbecco’s modified Eagle’s medium (DMEM) (10567014, Gibco) containing 20% fetal bovine serum (Gibco, 26140–079), maintained at 37 °C in a humidified atmosphere (5% CO_2_ in air), and grown to confluence. Cells were used between passages P1–P5.

### Leiomyoma cell culture and transfection

ht-UtLM cells tested negative for mycoplasma contamination were maintained in MEM (Sigma, #M3024) supplemented with 20% fetal bovine serum, 1 × MEM Vitamin Solution (Invitrogen #11120–052), 1 × MEM Amino Acids Solution (Invitrogen, #11130–051), and 1 × MEM Non-Essential Amino Acids (Invitrogen #11140–050). Primary UtLM-1 and UtLM-2 cells were maintained in DMEM (Gibco, #10567014) supplemented with 20% fetal bovine serum, and 1% penicillin/streptomycin, 1% amphotericin B. All cells were transfected in a 24-well plate scale. To prepare siRNA transfection solution for each well, 10 pmol (5 pmol for primary cells) of siCon, siH19, or siTET3 were mixed with 25 μl of OPTI-MEM by gentle pipetting. In parallel, 1 μl of Lipofectamine 3000 was mixed with 25 μl of OPTI-MEM. Following 5 min of incubation at room temperature, the two were mixed by gentle inverting and incubated for 10 min at room temperature, to allow siRNA/lipid complexes to form. At the end of incubation, the 50 μl of transfection solution was used to re-suspend the cell pellet (3 × 10^4^ cells). After incubation at room temperature for 10 min, growth medium was added at a ratio of 1:9 (1 volume of transfection solution/9 volumes of growth medium) and the cell suspension was transferred to a new culture dish. After overnight incubation in a tissue culture incubator, the medium was replaced with fresh growth medium. For iLet7 rescue experiments, 10 pmol of siCon/siH19 and 10 pmol of iCon/iLet7 were used for each well of 3 × 10^4^ cells. RNA, genomic DNA, and protein were extracted and analyzed at the indicated time points following transfection.

### Cell viability analysis

These were performed as previously described [30]. Briefly, cells were transfected and seeded in 96-well plates at a density of 4 × 10^3^/well. Cell viability and caspase-3/7 activity were measured 48 h post transfection using the CellTiter-Blue Cell Viability kit (Promega) and the Apo-ONE Homogeneous Caspase-3/7 Assay kit (Promega), respectively, according to the manufacturer’s protocols.

### Chromatin immunoprecipitation-quantitative PCR

These experiments were performed as previously described [35] with minor modifications. Briefly, experiments were performed in a 10 cm plate scale using the Pierce Agarose ChIP Kit (Thermo Scientific, 26156) according to the manufacturer’s instructions with minor modifications. Agarose beads were used to pre-bind overnight with antibodies against TET3 (Millipore Sigma, ABE290), H3K4me3 (Cell signaling, C42D8), and H3K27me3 (Cell signaling, C36B11). Pre-immune IgG was used as a negative control. Cells were cross-linked with 1% formaldehyde at room temperature for 10 min and the reaction was stopped by 1 × glycine. ChIPs were carried out overnight at 4 °C. Primers (Supplementary Table 2) for the specific promoter regions of *MED12*, *TGFBR2*, and *TSP1* (Fig. 3a) were used to amplify input and ChIP-purified DNA. The relative enrichments of the indicated DNA regions were calculated using the Percent Input Method according to the manufacturer’s instructions and were normalized to % input. Data are presented after normalization against background IgG signals.

### Quantitative methylation-specific PCR

gDNA was extracted from primary leiomyoma cells from 1 well of 24-well plates using Quick-gDNA MicroPrep (Zymo Research Corporation, Irvine, CA; D3021) according to the manufacturer’s instructions. For bisulfite treatment, 200 ng of gDNA was used for each column using EZ DNA Methylation-Gold Kit (Zymo, D5006). One hundred microliters of elution buffer was used to elute gDNA from each column. Real-time quantitative PCR was performed in a 15 μl reaction containing 5 μl of the eluted gDNA using iQSYBRGreen (Bio-Rad, Hercules, CA; 1708880) in a Bio-Rad iCycler. Two sets of PCR primers were designed: one for unmethylated and one for methylated DNA sequences. The PCR primers for methylated DNA were used at a final concentration of 0.6 μM in each PCR reaction. PCR was performed by initial denaturation at 95 °C for 5 min, followed by 40 cycles of 30 s at 95 °C, 30 s at 60 °C, and 30 s at 72 °C. Specificity was verified by melting curve analysis and agarose gel electrophoresis. The threshold cycle (Ct) values of each sample were used in the post-PCR data analysis. The relative levels of methylated vs. unmethylated DNA sequences are presented. The primers used for QMSP are listed in Supplementary Table 2.

### Statistical analysis

Statistical analyses and figure construction were performed using GraphPad Prism version 7.01 for Windows (GraphPad Software, La Jolla, California USA, www.graphpad.com). Data are analyzed using two-tailed Student’s *t*-test (or indicated otherwise in the figure legends) and presented as mean ± SEM. *P*-values at 0.05 or smaller were considered statistically significant.

## Supplementary information

Supplementary information

Supplementary Table 2

Supplementary Table 1
