# Supplementary material for: H19 lncRNA identified as a master regulator of genes that drive uterine leiomyomas
Source: Oncogene. 2019 May 15;38(27):5356–66. doi: 10.1038/s41388-019-0808-4 (PMC6755985; doi:10.1038/s41388-019-0808-4)
Supplement: Supplementary file 1 — Supplementary information [file 41388_2019_808_MOESM1_ESM.docx]

**Supplementary Figures and Methods**

**H19 lncRNA identified as a master regulator of genes that drive uterine leiomyomas**

Tiefeng Cao^1,2^*, Ying Jiang^1,3^*, Zhangsheng Wang^1,4^*, Na Zhang^5^, Ayman Al-Hendy^6^, Ramanaiah Mamillapalli^1^, Amanda N. Kallen^1^, Pinar Kodaman^1^, Hugh S. Taylor^1^, Da Li^7^, Yingqun Huang^1^


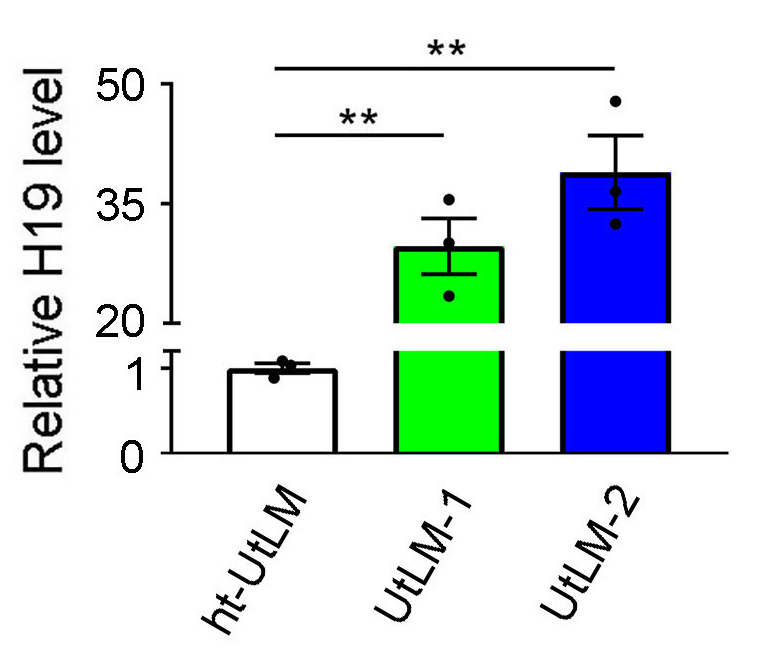


**Supplementary Fig 1**. RT-qPCR results of H19 expression from indicated leiomyoma cells. n=3, One-way ANOVA with Dunnett post-test. Data are representative of two independent experiments and are presented as mean ± SEM. **p < 0.01.


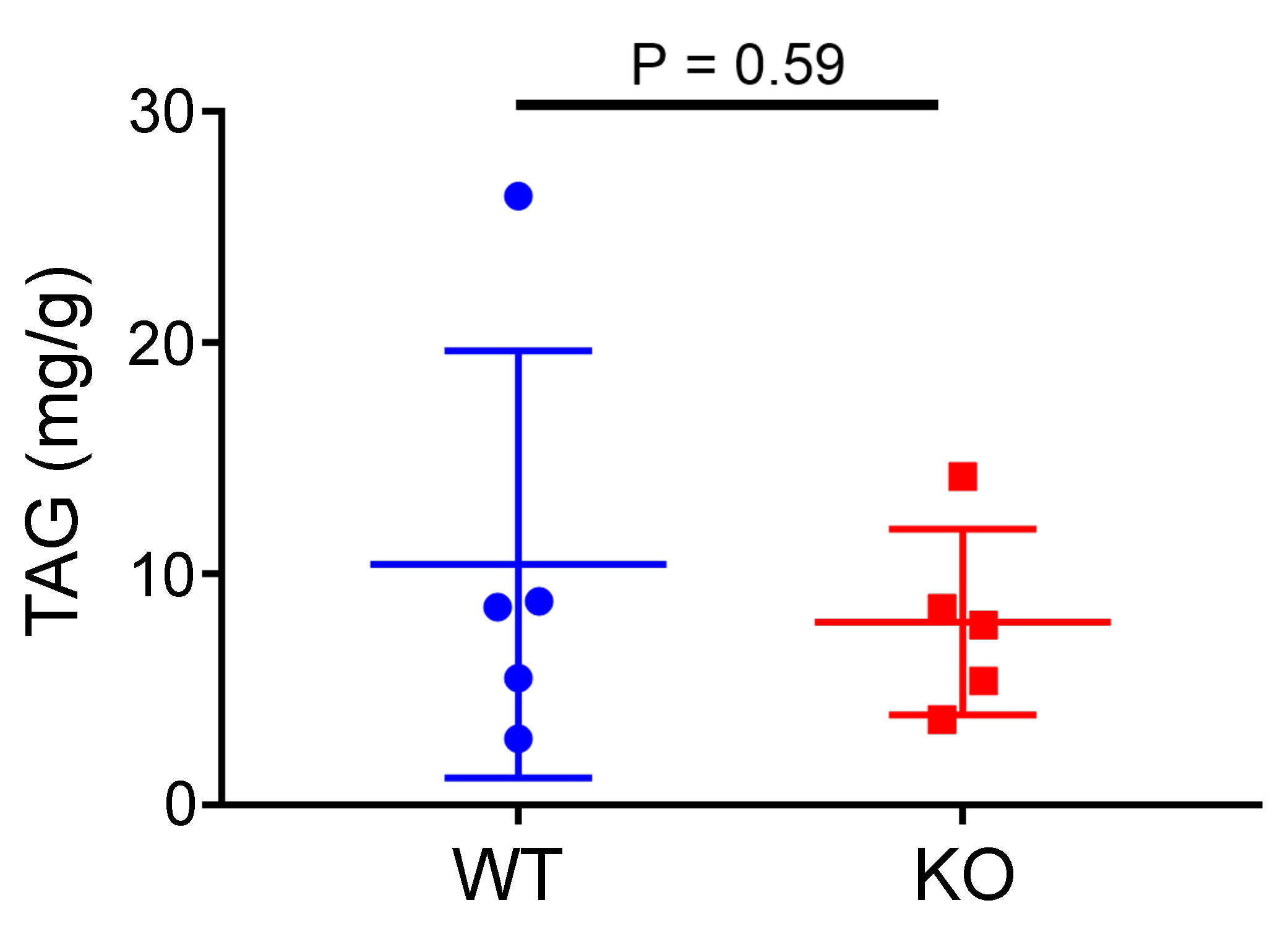


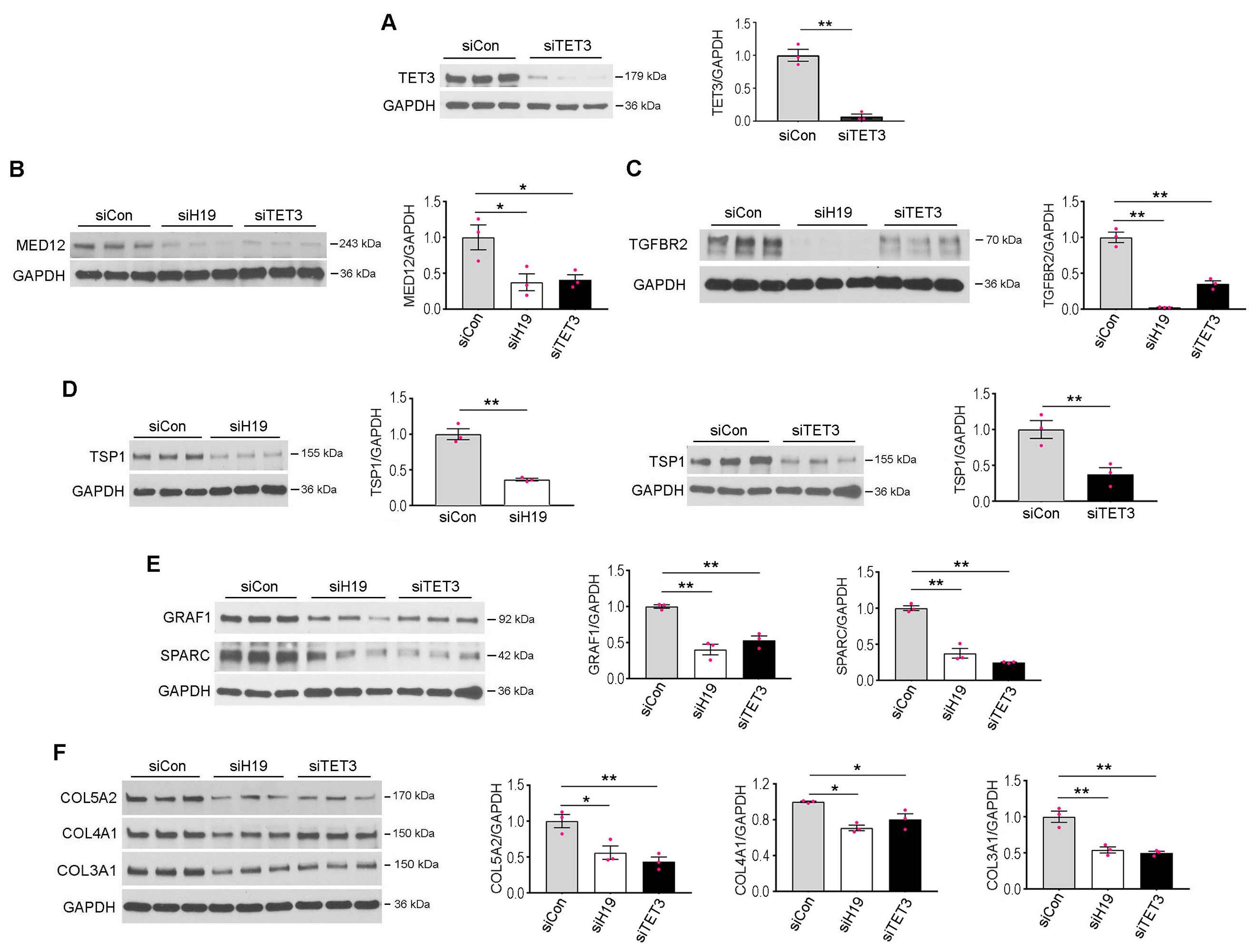


**Supplementary Fig 2. H19 and TET3 enhance fibroid-promoting gene expression at the protein level.** UtLM cells were transfected with siCon, siH19, or siTET3. Proteins were isolated 48 h later and levels were determined by Western blot analysis using GAPDH as a loading control. In **D**, TSP1 protein levels in the siCon/siH19 group were analyzed at 72 h post-transfection because no TSP1 protein level change was detected at the 48 h-time point (data not shown). The molecular sizes of the proteins are marked; quantifications of the protein bands are presented on the right sides of the gels. Each sample was loaded in triplicate. n=3. Data are representative of two independent experiments and are presented as mean ± SEM. *p < 0.05; **p < 0.01. **B**, **C**, **E**, **F**, One-way ANOVA with Dunnett post-test.

Similar observations were obtained using siTET3-b (Ambion, 4392420/s47238) targeted to a different region of human TET3 (data not shown).

**RNA extraction and RT-qPCR**

Total RNAs were extracted from cultured cells (ht-UtLM, UtLM-1, and UtLM-2) using PureLink RNA Mini Kit (Ambion, catalog number 12183018A). cDNA was synthesized using PrimeScript RT Reagent Kit (TAKARA, RR037A) in a 20 μl reaction containing 0.5 - 1 μg of total RNA. Real-time quantitative PCR was performed in a 15 μl reaction containing 0.5-1 μl of cDNA using iQSYBRGreen (Bio-Rad) in a Bio-Rad iCycler. PCR was performed by initial denaturation at 95°C for 5 min, followed by 40 cycles of 30 sec at 95°C, 30 sec at 60°C, and 30 sec at 72°C. Specificity was verified by melting curve analysis and agarose gel electrophoresis. The threshold cycle (Ct) values of each sample were used in the post-PCR data analysis. Gene expression levels were normalized against GAPDH. Real-time PCR primers are listed in Supplementary Table 2.

**Western blot analysis**

Primary leiomyoma cells grown in 24-well plates were detached by 0.25% trypsin digestion. After centrifugation at 1000 rpm for 5 min, supernatant was discarded and cell pallet was quickly homogenized in 2xSDS-sample buffer (100 μl/well of cells), followed by heating at 100 °C for 5 min with occasional vortexing. To prepare protein lysates from fibroids and normal myometrium tissue samples, 5 mg of fresh tissues were minced and 200 μl of 2xSDS-sample buffer was added, followed by homogenization on ice using a sonication machine. Homogenized samples were heated at 100 °C for 5 min and then centrifuged at 12,000g for 5 min to remove insoluble materials before loading onto 12% SDS gels (5 μl/well), followed by Western blot analysis. Bands on Western blot gels were quantified using ImageJ. GAPDH was used as a loading control.

**Genomic DNA extraction**

Genomic DNA (gDNA) was isolated using Quick-gDNA MicroPrep (Zymo, D3021) according to the manufacturer’s instructions.

**RNA-seq and data analysis**

Primary human uterine smooth muscle cells (UtSMC, CC-2562, Lonza) were purchased and maintained in culture media provided by the SmGM-2 Smooth Muscle Cell growth Medium-2 BulletKit (CC-3182, Lonza).

Cells were transfected with siCon or siH19 in a 6-well plate. Cells were harvested for RNA extraction at 48 h after transfection using the Purelink RNA mini kit (Ambion,12183018A). RNA-seq libraries were prepared using the Illumina TruSeq Stranded Total RNA LT kit with Ribo-Zero Human/Mouse/Rat, setA (rs-122–2201) according to the sample preparation protocol. Briefly,1 μg of total RNA was subjected to Ribo-Zero depletion to remove ribosomal RNAs (rRNAs). The remaining RNA was purified, fragmented, and primed with random hexamers for cDNA synthesis. After first and second cDNA synthesis, cDNA fragments were adenylated and then ligated to indexing adapters. The cDNA fragments were enriched by PCR, purified, and then sequenced on an Illumina NextSeq500 using paired-end chemistry and 76-bp cycles. Sequences are available from the GEO with accession number of GSE110557. Trophat v2.1.1 was used to map sequencing reads to hg38 genome. FeatureCounts v1.5.0 was used to estimate read counts for each gene. DESeq2 v1.14.1 was applied to calculate differential expression of genes.

**Methyl-MiniSeq library construction**

Libraries were prepared from 200 to 500 ng of gDNA digested with 60 units of TaqαI and 30 units of MspI (NEB) sequentially and then extracted with Zymo Research (ZR) DNA Clean & Concentrator-5 kit (Cat#: D4003). Fragments were ligated to pre-annealed adapters containing 5′- methyl-cytosine instead of cytosine according to Illumina’s specified guidelines (www.illumina.com). Adaptor-ligated fragments of 150–250 and 250–350 bp in size were recovered from a 2.5% NuSieve 1:1 agarose gel (Zymoclean Gel DNA Recovery Kit, ZR Cat#: D4001). The fragments were then bisulfite-treated using the EZ DNA Methylation-Lightning Kit (ZR, Cat#: D5020). Preparative-scale PCR was performed and the resulting products were purified (DNA Clean & Concentrator-ZR, Cat#D4005) for sequencing on an Illumina HiSeq.

**Methyl-MiniSeq sequence alignments and data analysis**

Sequence reads from bisulfite-treated EpiQuest libraries were identified using standard Illumina base-calling software and then analyzed using a Zymo Research proprietary analysis pipeline, which is written in Python and uses Bismark (http://www.bioinformatics.babraham.ac.uk/projects/bis mark/) to perform the alignment. Index files were constructed using the bismark_genome_preparation command and the entire reference genome. The non-directional parameter was applied while running Bismark. All other parameters were set to default. Filled-in nucleotides were trimmed off when doing methylation calling. The methylation level of each sampled cytosine was estimated as the number of reads reporting a C, divided by the total number of reads reporting a C or T. Fisher’s exact test or t-test was performed for each CpG site which has at least five reads coverage, and promoter, gene body and CpG island annotations were added for each CpG included in the comparison. Methylation data are available at GEO accession GSE117190.
