# Supplementary material for: H19 lncRNA identified as a master regulator of genes that drive uterine leiomyomas
Source: Oncogene. 2019 May 15;38(27):5356–66. doi: 10.1038/s41388-019-0808-4 (PMC6755985; doi:10.1038/s41388-019-0808-4)
Supplement: Supplementary file 2 — Supplementary Table 2 [file 41388_2019_808_MOESM2_ESM.docx]

| **Real-time PCR primer sequences for human genes** | | |
| --- | --- | --- |
| **Gene** | **Forward Primer** | **Reverse Primer** |
| H19 | 5’-ACTCAGGAATCGGCTCTGGAA-3’ | 5’-CTGCTGTTCCGATGGTGTCTT-3’ |
| TET3 | 5’-GACGAGAACATCGGCGGCGT-3’ | 5’-GTGGCAGCGGTTGGGCTTCT-3’ |
| MED12 | 5’-GCCATGAGTGCAACCAGCGG-3’ | 5’-ATGTCAGGTCTCCAGGATTCAG-3’ |
| TGFBR2 | 5’-GGTTCCTGTGTGCCCTTATT-3’ | 5’-TGCAACCCATGAAGGTAAAA-3’ |
| THBS1 | 5’-AGCGTCTTCACCAGAGACCT-3’ | 5’-CATTCACCACGTTGTTGTCA-3’ |
| GRAF1 | 5’-ACCTGCACTTGCTTTTTGAC-3’ | 5’-CCACGTAATTCTCAGGGATG-3’ |
| SPARC | 5’-GCTGGATGAGAACAACAC-3’ | 5’-AAGAAGTGGCAGGAAGAG-3’ |
| COL3A1 | 5’-CTGGAACAAGTGATGCCTCT-3’ | 5’-CATGCAGACATTACGACCAA-3’ |
| COL4A1 | 5'- TCCATACTGTTTGCCCATTT-3’ | 5'- TCCATTTGGAGGTTCAAAAA-3’ |
| COL5A2 | 5'- TTAAATGCACGCTTTTGTCA -3’ | 5'- TTGCCTTTGTGGGTAATGTT-3’ |
| GAPDH | 5’-CTTTGTCAAGCTCATTTCCTGG-3’ | 5’-TCTTCCTCTTGTGCTCTTGC-3’ |

| **ChIP-PCR primer sequences** | | |
| --- | --- | --- |
| **Gene** | **Forward** | **Reverse** |
| MED12 | 5’-AGTCAGCCTGGCCCTGCT-3’ | 5’-AACGCACCTCCTTCTGTTTGGG-3’ |
| TGFBR2 | 5’-GGGCTGGTCTAGGAAACATGATTGG-3’ | 5’-GAAACAGGAAACTCCTCGCCAACA-3’ |
| TSP1 | 5’-CCCATTGGCCGGAGGAATCCC-3’ | 5’-GGCTGGCAAGGCGGAGGAG-3’ |

| **QMSP primer sequences** | | |
| --- | --- | --- |
| **Gene** | **Forward Primer** | **Reverse Primer** |
| MED12 methylated | 5’-AACGTGTTTTTTTGTTGTTTTTCG-3’ | 5’-CACCTCCTTCTATTTAAAATCCTAAAAA-3’ |
| MED12 unmethylated | 5’-GTTTTTAATGTGTTTTTTTGTTGTTTTTTG-3’ | 5’-CACCTCCTTCTATTTAAAATCCTAAAAA-3’ |
| TGFBR2 methylated | 5’-GGAGAGGGAGAAGGTTTTCG-3’ | 5’-AATAACTCACTCAACTTCAACTCAAC-3’ |
| TGFBR2 unmethylated | 5’-AGGAGAGGGAGAAGGTTTTTG-3’ | 5’-AATAACTCACTCAACTTCAACTCAAC-3’ |
| TSP1 methylated | 5’-AGAAGAAAAAAAAAATTTAATTATTTTTCG-3’ | 5’-CAAATCATAAATAATAATTCATCCAAACA-3’ |
| TSP1  unmethylated | 5’-GAAAGAAGAAAAAAAAAATTTAATTATTTTTTG-3’ | 5’-CAAATCATAAATAATAATTCATCCAAACA-3’ |
